# Prioritizing Built Environmental Factors to Tackle Chronic and Infectious Diseases in Remote Northern Territory (NT) Communities of Australia: A Concept Mapping Study

**DOI:** 10.3390/ijerph18105178

**Published:** 2021-05-13

**Authors:** Amal Chakraborty, Natasha J. Howard, Mark Daniel, Alwin Chong, Nicola Slavin, Alex Brown, Margaret Cargo

**Affiliations:** 1 School of Health Sciences, University of South Australia, Adelaide, SA 5001, Australia; natasha.howard@sahmri.com (N.J.H.); mark.daniel@canberra.edu.au (M.D.); alex.brown@sahmri.com (A.B.); margaret.cargo@canberra.edu.au (M.C.); 2 Research Centre for Palliative Care, Death and Dying, College of Nursing and Health Sciences, Flinders University, Bedford Park, SA 5042, Australia; 3 Wardliparingga Aboriginal Health Equity, South Australian Health and Medical Research Institute, Adelaide, SA 5000, Australia; 4 Adelaide Medical School, Faculty of Health and Medical Sciences, University of Adelaide, Adelaide, SA 5000, Australia; 5 Health Research Institute, Faculty of Health, University of Canberra, Bruce, ACT 2601, Australia; 6 South Australian Health and Medical Research Institute, Adelaide, SA 5000, Australia; 7 Australian Centre for Child Protection, University of South Australia, Adelaide, SA 5001, Australia; marawuy@bigpond.com; 8 Environmental Health Branch, Department of Health, Northern Territory Government, Casuarina, NT 0810, Australia; nicola.slavin@nt.gov.au

**Keywords:** indigenous populations, public health, environmental health, built environment, housing, environmental indicators, communicable diseases, chronic diseases, public policy, perception

## Abstract

High prevalence of chronic and infectious diseases in Indigenous populations is a major public health concern both in global and Australian contexts. Limited research has examined the role of built environments in relation to Indigenous health in remote Australia. This study engaged stakeholders to understand their perceptions of the influence of built environmental factors on chronic and infectious diseases in remote Northern Territory (NT) communities. A preliminary set of 1120 built environmental indicators were systematically identified and classified using an Indigenous Indicator Classification System. The public and environmental health workforce was engaged to consolidate the classified indicators (*n* = 84), and then sort and rate the consolidated indicators based on their experience with living and working in remote NT communities. Sorting of the indicators resulted in a concept map with nine built environmental domains. Essential services and Facilities for health/safety were the highest ranked domains for both chronic and infectious diseases. Within these domains, adequate housing infrastructure, water supply, drainage system, reliable sewerage and power infrastructure, and access to health services were identified as the most important contributors to the development of these diseases. The findings highlight the features of community environments amenable to public health and social policy actions that could be targeted to help reduce prevalence of chronic and infectious diseases.

## 1. Introduction

The World Health Organisation (WHO) estimates that about 22% of the global burden of disease, and 23% of all deaths are attributable to modifiable environmental factors [1]. Disease burdens associated with environmental exposures are largely the result of chronic (e.g., cardiovascular) and infectious (e.g., respiratory) diseases. The WHO calls for creating and maintaining healthful environments as a priority for primary prevention [1]. This call is supported by the Aboriginal and Torres Strait Islander (hereafter respectfully referred to as Indigenous) leadership [2,3], who have been advocating for improvements in environmental living conditions due to the lowest life expectancy and greater disease burden in Australian Indigenous communities [4,5].

The gaps in life expectancy, mortality and disease burdens between Indigenous and non-Indigenous Australians are primarily driven by preventable chronic disease and infectious disease [6,7]. Indigenous people living in remote areas, particularly in the Northern Territory (NT) of Australia, experience poorer health outcomes compared to Indigenous people living in other Australian states and territories [8,9]. The high prevalence of chronic and infectious diseases in remote Indigenous communities is deemed to have resulted from the interaction between Indigenous peoples’ exposure to rapid environmental changes following the discriminatory policies and actions relating to colonization [10,11]. Indigenous peoples’ exposure to a westernized way of living in a changed environment was associated with the adoption of unhealthful lifestyle behaviors including smoking, physical inactivity and alcohol consumption [12].

So far, existing research has tended to attribute the development of chronic disease and infectious disease in Indigenous Australians to individual-level behavioural ‘risk factors’ [9,13,14,15]. Studies generally overlook that individual-level behavioural ‘risk factors’ may reflect underlying collective exposure to ‘risk conditions’—that is, unfavourable built, social, cultural and political environments [12]. This study was grounded on the notion that the features of the places where people live, particularly built environmental living conditions, and the opportunities these places provide for making healthy choices, shape people’s health behaviour, and thus the risk factors for chronic disease and infectious disease more broadly [16,17].

A growing body of research has measured the effects of built environmental factors on chronic disease and infectious disease outcomes in non-Indigenous populations [18]. Studies have identified that features of built environments such as poor sports and recreational facilities, limited opportunities for walking and cycling, automobile use and exposure to indoor allergens can increase the risk of chronic disease [12,19,20]. Evidence also suggests, that healthful residential public open space (e.g., greenness) can protect against psychological distress [21]; by contrast, dwelling density, proximity to fast food outlets, unhealthful public open space can exacerbate Body Mass Index (BMI) and waist circumference [22]. It is acknowledged that elements of the built environment vary by community context and can influence health via different biological, behavioural and psychosocial pathways in both Indigenous and non-Indigenous populations [12,23,24].

Limited research has investigated associations between the built environment and Indigenous health, particularly for remote communities in the NT. Of the research that has been conducted, most has characterized aspects of housing-related built environment infrastructure and infectious disease [25,26]. These studies report that housing issues such as overcrowding, unreliable running water and power supply, non-functional sewerage facilities and inadequate waste disposal greatly impact health, particularly infectious diseases, of Indigenous communities.

Given that infectious diseases, if not treated early in their onset, contribute to the development of chronic diseases, it is essential to examine both chronic disease and infectious disease in relation to remote community environmental living conditions [27,28]. Inadequate and unsanitary living conditions directly and indirectly influence the spread of bacterial and non-bacterial infections [29]. Frequently observed infections in remote Indigenous communities are generally transmitted by physical person-to-person contact, fomites (transmission of infection by objects, when viruses or bacteria remain on surfaces), and through animals in highly contaminated home or community built environments. Improving built environmental living conditions can play an important role during disease outbreaks, such as the coronavirus, SARS-CoV-2 (COVID-19), impacting compliance with the public health measures including hand washing, physical distancing and isolation of infectious disease cases [30].

Federal, state and territory governments in Australia fund strategic programs and services to improve the environmental living conditions in Indigenous communities to reduce the disparities in health outcomes. As part of the ‘Closing the Gap’ strategy, all levels of government use a range of indicators to monitor and evaluate the performance and functioning of essential services and community infrastructure. Despite a call for evidence-based policy making to ensure transparency and accountability in funding management for program and service delivery in remote Indigenous communities [31], it is not clear which remote built environmental living conditions should be prioritized for ongoing monitoring and surveillance by environmental and public health authorities.

The primary aim of this study was to engage Indigenous and non-Indigenous public and the environmental health workforce members (i.e., frontline, managerial and policy-level staff) in a strategic planning process to prioritize built environmental factors that they deem to influence the development of chronic disease and infectious disease in remote Indigenous communities in the NT, Australia. Using a participatory mixed-method Group Concept Mapping (GCM) research approach the objectives of this study were to:

(1) consolidate built environmental indicators relevant to the development of chronic disease and infectious disease;
(2) engage stakeholders to sort a set of consolidated built environmental indicators into conceptually meaningful groupings;
(3) engage stakeholders to rate a set of consolidated built environmental indicators on perceived importance in relation to their influence on chronic disease and infectious disease; and
(4) engage stakeholder organizations in the interpretation and utilization of the results to further inform environmental public health practice in remote Indigenous communities in the NT.

## 2. Materials and Methods

### 2.1. Study Context

This GCM study was nested within a four-year Australian National Health and Medical Research Council (NHMRC) funded project grant titled ‘Environments and Remote Indigenous Cardiometabolic Health (EnRICH)’. The EnRICH Project aimed to evaluate features of social, built and physical (e.g., temperature) environments in relation to community-level cardiometabolic disease outcomes in 123 remote Indigenous communities of the NT [23]. With an area of approximately 1,348,094 square kilometers, and covering one-sixth of the Australian landmass, the NT is the third-largest Australian federal division. The vast majority of the NT landmass is classified as ‘remote’ following the Australian Standard Geographical Classification (ASGC) Remoteness Area [32]. According to the 2016 Census of Population and Housing [33], there were 228,833 people living in the NT, of which 58,248 people (25.5%) were Indigenous. Approximately 80% of the total Indigenous population living in the NT, lived in a remote or very remote area [33].

### 2.2. Research Approach

This GCM study undertook an integrated knowledge translation (iKT) approach, identified as best practice for population and public health research [34,35]. A working group was formed, comprising seven frontline and policy-level Indigenous (*n* = 2) and non-Indigenous (*n* = 5) members from the public and environmental health workforce with experience living and/or working in remote communities, and its intersection with environmental and public health policy and practice in the NT. Decision makers and frontline staff were viewed as experts on the local environmental living conditions affecting the health of Indigenous people living in remote communities. Having senior project officers and policy level decision makers in the working group contributed to the concept mapping study in several ways: (1) making the research protocol relevant to the local context; (2) obtaining organizational support; (3) brokering participation of the frontline workforce; and (4) facilitating the interpretation and translation of the results, to inform areas of focus for public and environmental public health policy and practice.

Engagement with government and not-for-profit stakeholder organizations in the NT suggested that the project needed to focus on built environmental factors in relation to the development of both chronic and infectious diseases. Initially the focus of this study was on chronic disease. Therefore, the research responded to identified stakeholder concerns, and was expanded to include infectious disease.

In order to ensure cultural integrity, an Indigenous cultural mentor (A.CH.) was engaged as part of the study team who provided cultural oversight throughout the research process, from identification of preliminary set of built environmental indicators to data analysis and interpretation of concept maps. A.CH., a Wakamin man from Northern Queensland, Australia has extensive experience in research and teaching in the Indigenous health and social policy sector. A.CH. is also a respected Aboriginal elder, and he is well known nationally for his advocacy and dedication to Indigenous health and health research.

### 2.3. Study Design: Concept Mapping

GCM methodology was selected as it engages stakeholders in a systematic and transparent approach to identifying, as well as prioritizing strategies on a topic of practical and/or policy relevance to facilitate knowledge translation [36]. This mixed-method approach has evolved over a period of nearly 35 years and has been widely applied in public health, environmental health, and program evaluation [37,38]. GCM utilizes both qualitative and multivariate statistical techniques to represent stakeholders’ ideas visually in a series of interpretable two-dimensional maps [39]. This GCM study followed the procedures outlined by Kane and Trochim [39], and drew on some of the research team members’ prior experience with application of the methodology in Indigenous and non-Indigenous context in Australia and New Zealand [40,41,42]. The study was implemented in a six-step sequential process comprising preparation, brainstorming, sorting and rating, analysis, interpretation, and utilization of the results [39].

#### 2.3.1. Preparation

The preparation step involved developing the research question and study materials, identifying and recruiting participants, arranging logistics for stakeholder participation, and obtaining ethical approval. A forward statement, a focus prompt, and two perceived importance rating questions were developed to guide the study. A set of demographic questions was developed covering areas of work, type of organization, ethnicity/ancestry, years of work experience in Indigenous communities, and position/role. The preparation activities were reviewed and refined by the Indigenous cultural mentor, and the working group members who also facilitated obtaining organizational support prior to inviting participants into the study.

This study sought to engage Indigenous and non-Indigenous frontline workers, as well as managerial and policy-level staff members, who worked in, or had responsibility to provide, environmental and/or public health services in one or more of the identified remote Indigenous communities in the NT. The types of roles or positions of the participants involved in this research included: Environmental Health Officers, Primary Health Care Workers, Program Development Officers, Project Officers, Team Leaders and Managers. These workforce members were identified as having the greatest knowledge of remote community environments, and associated built environmental indicators in the NT. Although all participants had experience delivering services in remote communities, some participants were responsible for providing public and environmental health services to rural and urban communities as well.

Multiple strategies were used to recruit participants, including utilizing existing relationships with the EnRICH Project stakeholder organizations. A number of government and Indigenous community-controlled health organizations working in the areas of public and environmental health in the NT were invited to participate in the research project. These included a state-level government environmental health agency, a not-for-profit veterinary and health promotion organization, a community-controlled infrastructure and technology organization, a community-controlled health organization, two regional offices of a federal government department, and a regional council. Obtaining organizational support was necessary prior to inviting participants into the study. This required travel to different forums and meetings in Darwin, Alice Springs and Katherine of the NT to explain the research project and benefits of participation to key stakeholder organizations. As relationships with organizations were developed, and organizational support was obtained, their Indigenous and non-Indigenous staff members were then invited to participate in the study. Potential participants were subsequently emailed an information sheet, and consent form, with supporting materials relevant to the study.

Concept System Global Max (version 4.0, Concept Systems Incorporated, Ithaca, NY, USA) software was used to engage participants online, and to manually enter data that were collected from face-to-face sorting and rating sessions. 

#### 2.3.2. Brainstorming

The brainstorming activity in this GCM study was informed by a preceding scoping review of community-level strategic planning documents [43]. A group of experienced Indigenous (A.CH., and A.B.) and non-Indigenous researchers (A.C., N.J.H., M.D., N.S., and M.C.) with expertise in community engagement and Indigenous culture, spatial epidemiology, health promotion, and prevention of chronic disease and infectious disease guided the identification of documents, and extraction and synthesis of built environmental indicators as part of the scoping review. An established Indigenous Indicator Classification System (IICS) [44,45] was integrated in the scoping review [43] to guide the classification, measurement and validation of existing built environmental indicators from Indigenous community members’ perspectives. The IICS was adapted from the “German System of Social Indicators” through a collaboration of Indigenous and non-Indigenous researchers, and community stakeholders from Canada, Australia and New Zealand [44,45].

Following structured statement synthesis process [39], undertaken by the first author, the classified indicators generated from the scoping review were consolidated into common themes (*n* = 105) and then transformed into statements (*n* = 84) for subsequent sorting and rating activities. The final pool of common themes and statements were reviewed and refined by the working group. The brainstorming activity was guided by the following focus prompt:

*A list of built environmental living conditions that contribute to the development of preventable chronic disease and infectious disease in remote Indigenous communities is given below. This information is taken and summarized from the publicly available planning documents that were developed through community consultations. Please review the following list and refine/add further built environmental living conditions that you think contribute to the development of chronic disease and infectious diseases in Indigenous communities where you live and/or work* ….

#### 2.3.3. Sorting

Participants who provided voluntary informed consent, completed the sorting tasks either face-to-face or online. The face-to-face sorting exercise was generally held in a group workshop setting but participants were asked to complete the task individually, and not to consult with one another. Participants who chose to complete the sorting on-line were sent a sorting web-link for the Concept Systems Global Max software hosted within the http://www.conceptsystems.com/ website (accessed on 12 May 2021). Upon entering the Concept Systems website via the link, participants were guided to the project introduction page to re-read the participant information sheet, and then self-register/sign-up by creating a user profile. The project home page included further instructions, including a number of chronological activities to be conducted. Sorting the indicator statements online required following the same instructions as the face-to-face exercise for grouping and labelling of the piles.

Following the recommended procedure [39], participants were asked to group all the 84 consolidated indicator statements into separate piles according to their perceived similarity. Four key rules were applied in sorting the statements: (1) the statements cannot be placed into just one pile; (2) each statement cannot form a pile on its own; (3) dissimilar (unrelated) statements cannot be grouped together in one pile (i.e., a miscellaneous pile); and (4) sorting the statements into a minimum of 8 piles and maximum of 20 piles was recommended.

#### 2.3.4. Rating

The rating activity involved the same participant groups as those of the sorting activity. Stakeholder participants who provided voluntary informed consent also completed the rating tasks either face-to-face or online. The face-to-face rating exercise was generally held in a group workshop setting on the same day that the participants completed the sorting tasks. Similar to the on-line process as described above for sorting, following the receipt of consent for participation, those participants who chose to complete the rating online were sent a rating web-link for the Concept Systems Global Max software hosted within the http://www.conceptsystems.com/ website (accessed on 12 May 2021).

Two sets of rating sheets were produced by the authors (A.C. and N.J.H.) and distributed to participants that contained the same 84 indicator statements on each, with one set under the category of ‘chronic disease’, and the other under ‘infectious disease’. Participants were asked to rate the statements relative to each other using a 5-point scale, representing 1 = not at all important, and 5 = extremely important. Participants were also reminded that they needed to consider the full range of the 5-point scale when rating the indicators. The prompts reported below guided assignment of the ratings.

*Rating activity—chronic disease: For Aboriginal and Torres Strait Islander people living in remote communities, please rate on a scale of 1 to 5 how important each of the following statements are in relation to their influence on* chronic disease (e.g., type 2 diabetes, kidney disease, heart disease). *The influence of the environment on chronic disease can be direct or indirect.*

*Rating activity—infectious disease: For Aboriginal and Torres Strait Islander people living in remote communities, please rate on a scale of 1 to 5 how important each of the following statements are in relation to their influence on* infectious disease (e.g., skin infection, ear infection, respiratory infection). *The influence of the environment on infectious disease can be direct or indirect.*

#### 2.3.5. Analysis

Data generated from the sorting and rating activities was entered into Concept Systems Global Max software for analysis. A unique binary matrix of similarities was created based on the sort information from each participant. This binary similarity matrix was the input for non-metric multidimensional scaling (MDS) analysis which represents the dis(similarity) of the statements in terms of distance in Euclidean space. In MDS analysis, each statement has an assigned *x* and *y* value resulting in a bivariate plot of coordinates generating a point map [46]. The indicator statements grouped together frequently in the sorting activity ended up positioned closer to each other. At this stage, a stress index was calculated to provide a diagnostic estimate indicative of how well the data from the similarity matrix fit the point map. Stress values range from 0 (perfect fit) to 1 (worse fit), with an index between 0.205 and 0.365 considered to demonstrate a good fit for a field based concept mapping study [47].

The *x* and *y* coordinates from the MDS analysis were used as input for the hierarchical cluster analysis which partitioned the points on the map into non-overlapping clusters. Although there is no strict mathematical criterion to determine the optimal cluster solution, selection of the number of clusters can be influenced by the anchoring of statements on the map, statement and cluster bridging values, stress index, and non-overlapping conditions of the cluster solution. A review of concept mapping studies reported a median cluster solution of about 9 clusters and range of 6 to 14 clusters [48]. Further, bridging values ranging from 0 to 1 were calculated for each statement and cluster. A lower bridging value (e.g., 0.10) indicates a ‘tighter’ relationship between the statements within each cluster and a close association to specific areas of the map compared to statements with higher bridging values (e.g., 0.81).

For the rating data, average importance ratings were computed separately for chronic disease and infectious disease across all statements within clusters and visually displayed in a bivariate ladder graph (pattern matching). Independent sample t-tests and confidence intervals (CI 95%) of importance ratings were calculated for the assessment of statistically significant differences between the clusters.

A four quadrant ‘Go-Zone’ map, which visually depicts the importance relationships at the indicator statement level, was produced. The coordinates for each statement were plotted as a point in one of the four quadrants. Each point in a ‘Go-Zone’ map represents the average rating for each indicator statement in the cluster. The name ‘Go-Zone’ reflects the top right-hand green quadrant on the map illustrating the statements of greatest importance or highest priority in relation to both chronic and infectious diseases, depicted by greater-than-average importance scores given by the participants.

#### 2.3.6. Interpretation and Utilization

In this study, stakeholders were engaged at the beginning to foster ownership and facilitate the utilization of the findings. Following the completion of data collection and analysis, the resultant concept maps were interpreted in three separate structured group sessions by stakeholder participants from frontline (*n* = 7) and managerial and policy (*n* = 4) workforce in public and environmental health in the NT. Any changes to the clusters or the map were made with consensus from the participants. Cluster labels and the optimal number of clusters in the solution were then finalized with stakeholder input.

## 3. Results

### 3.1. Participant Characteristics

Table 1 summarizes participant demographic characteristics for the sorting and rating activities. Of the 55 participants invited to participate, 29 participants completed the sorting task whereas, 42 participants completed the rating task. About 90% (*n* = 26) of the sorting participants were engaged face-to-face to sort the indicator statements. Similarly, about 88% (*n* = 37) of the rating participants were engaged face-to-face to rate the indicator statements. Remaining participants completed these activities online. The majority of participants, including nine Indigenous participants, were from the government public and environmental health sectors, with responsibility for covering predominantly remote geographic regions of the NT. Most participants identified as non-Indigenous, and had 6 years or more experience in their respective positions. Further, most participants represented the frontline workforce.

### 3.2. Sorting

**Figure 1:**
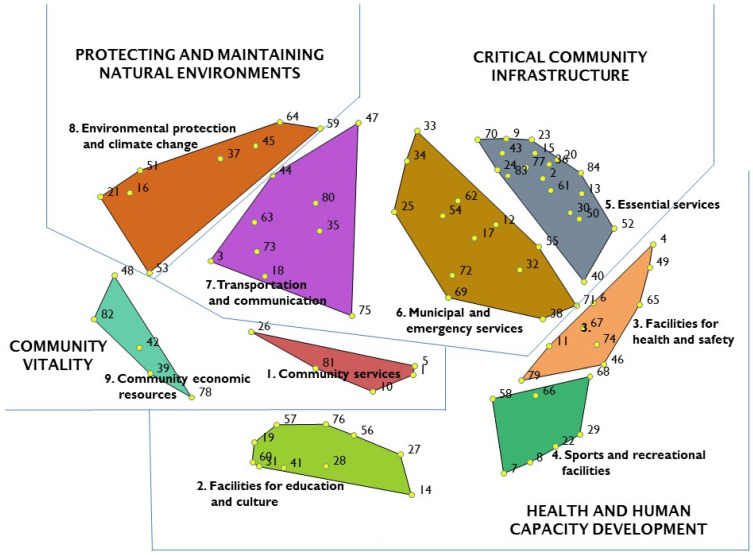
Final cluster map of built environments showing regions of similarity, name of clusters and indicator statement numbers (see Supplementary File: Table S1 for a description corresponding to each indicator statement number).

Based on stakeholder participant inputs, a nine-cluster concept map with a stress value of 0.313 was deemed the optimal solution in terms of sorting data and point distances between the 84 indicator statements (see Supplementary File: Table S1 for a description of each of the 84 indicator statements). During the sorting activity, the median number of piles created by individual participants was 10 (range 4–18). The concept map illustrated in Figure 1 depicts four overarching regions of similarity, which pertain to: (1) critical community infrastructure; (2) health and human capacity development; (3) community vitality; and (4) protecting and maintaining natural environments. The ‘essential services’ (bridging value 0.08; range 0.00–0.23) and ‘community economic resources’ (bridging value 0.81; range 0.71–1.00) clusters represent statements with the lowest and the highest average bridging values, respectively (see Supplementary File: Table S1). These bridging values reflect that indicator statements within the ‘essential services’ cluster were grouped together more frequently and are firmly anchored to their position on the map, while the statements within the ‘community economic resources’ cluster were frequently grouped together with statements which were not in their immediate vicinity.

### 3.3. Rating

The relative mean importance ratings across all participants (*n* = 42) for the 84 indicator statements and their corresponding 9 clusters are provided in Supplementary File: Table S1. The importance ratings in relation to chronic disease ranged from 1.71 to 4.83 (M = 3.31, SD = 0.73), and for infectious disease ranged from 1.90 to 4.88 (M = 3.29, SD = 0.81). At the cluster level, the aggregated average importance rating for all clusters in relation to chronic disease ranged from 2.51 to 4.02 (M = 3.21, SD = 0.47), and for infectious disease ranged from 2.39 to 4.12 (M = 3.13, SD = 0.55).

**Figure 2:**
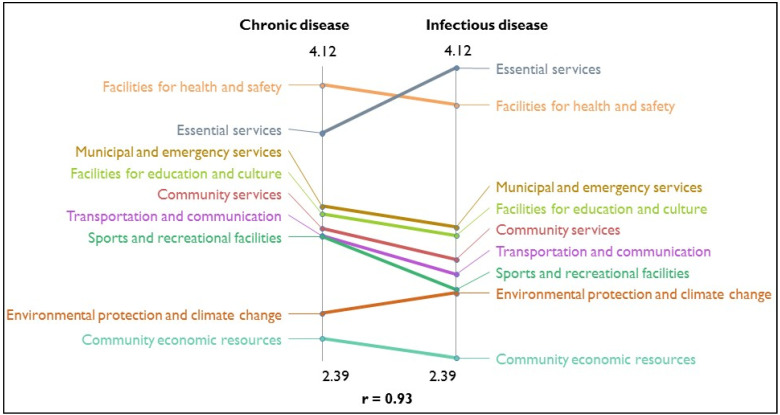
Ladder graph depicting absolute differences in mean importance ratings for chronic disease and infectious disease, by cluster.

The “ladder graph” or pattern match illustrates the level of agreement between average cluster ratings on perceived importance for chronic disease and infectious disease. Figure 2 depicts that the ‘essential services’ cluster was ranked the highest for infectious disease and the ‘facilities for health and safety’ cluster, ranked the highest for chronic disease. The correlation coefficient between the mean importance ratings at the cluster level was very strong (r = 0.93), demonstrating similarity in the average cluster ratings. An Independent Samples *t*-test indicated perceived mean importance ratings between *chronic disease* and *infectious disease* were not statistically significantly different.

**Figure 3:**
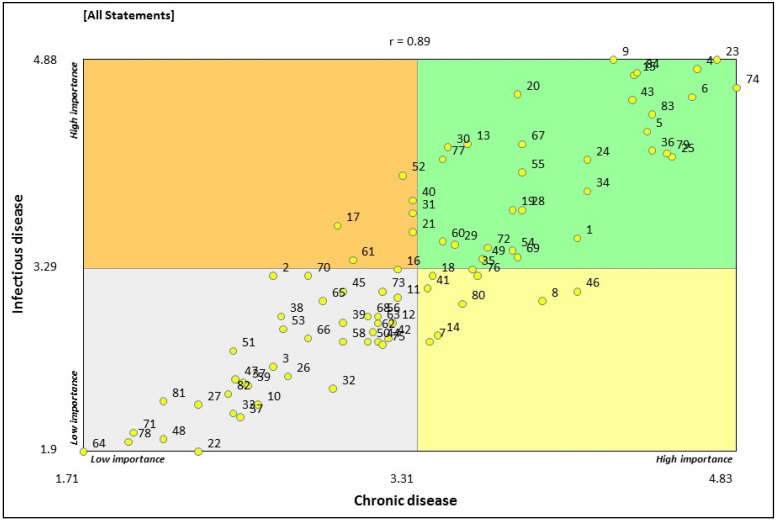
Bi-variate ‘Go-Zone’ plot of mean perceived importance ratings of indicator statements in relation to their influence on chronic disease and infectious disease.

Figure 3 illustrates of all 84 indicator statements, 30 were located within the green ’Go-Zone’. The overall correlation between the perceived importance ratings for both chronic and infectious disease was strong (r = 0.89). An additional analysis of relative representation of cluster statements (expressed as a proportion) suggests, four of the nine clusters had 40% or more of their indicator statements plotted in the green ‘Go-Zone’ (see Supplementary File Table S1). Approximately 67% of all statements within the ‘essential services’ and the ‘facilities for health and safety’ clusters were located within the green ‘Go-Zone’. The indicator statements within the ‘essential services’ cluster of highest importance, both for chronic and infectious diseases, were related to ‘access to power and water (statement ID# 15, 23, 36, and 83)’, ‘sewerage and septic system (ID# 9 and 84)’, ‘maintenance of housing infrastructure (ID# 43)’, and ‘regular rubbish collection (ID# 20)’. Most notable among these indicator statements is ‘access to continuous water supply’ (ID# 23)’ which received the highest average importance rating (i.e., 4.81). The indicator statements within the ‘facilities for health and safety’ of highest importance, both for chronic and infectious diseases, were related to ‘adequate housing (ID# 4)’, ‘hospital services (ID# 6)’, ‘community health services (ID# 74)’, and ‘aged care (ID# 79)’. Three clusters, i.e., ‘transportation and communication’, ‘environmental protection and climate change’, and ‘community economic resources’ did not have any indicator statements represented in the green ‘Go-Zone’.

## 4. Discussion

This GCM study was an innovative first effort to examine perceptions of the frontline, managerial and policy-level public and environmental health workforce on the importance of built environmental indicators in relation to their influence on chronic disease and infectious disease. Application of an iKT approach resulted in a nine-cluster solution concept map, with four regions (Figure 1): (1) critical community infrastructure, (2) health and human capacity development, (3) community vitality, and (4) protecting and maintaining natural environments. The key findings within each region, and their implications for Indigenous health and wellbeing, including COVID-19 disease outbreak, are highlighted below.

The ‘critical community infrastructure’ region comprised three clusters, of which the ‘essential services’ cluster ranked as the highest in perceived importance for infectious disease. Within the ‘essential services’ cluster, power supply (statement ID# 36, and 83), water and sanitation (ID# 9, 13, 15, 23, and 84), and housing maintenance (ID# 43) were perceived the most dominant. environmental living conditions. These core features of community infrastructure have important health implications for remote communities in the NT [49,50,51,52]. For instance, a reliable power supply ensures that remote community households can store their foods properly refrigerated to avoid the spread of infectious diseases such as gastrointestinal disease. A continuous supply of water supports good hygiene (e.g., washing hands, washing clothes) to protect children and family members from scabies and respiratory infections [53].

Similarly, the ‘health and human capacity development’ region comprised three clusters, of which the ‘facilities for health and safety’ cluster ranked as the highest in perceived importance for chronic disease. Within the ‘facilities for health and safety’ cluster, access to health services (ID# 6, 74, and 79), and adequate residential housing infrastructure (including housing condition and overcrowding) (ID# 4) were perceived the most dominant environmental living conditions. These features of community environment also have important implications for Indigenous health and wellbeing [52,54]. For example, the literature highlights the physical presence, accessibility, and utilization of primary, secondary and tertiary health services as a major contributor to chronic disease management. This health infrastructure allows Indigenous community members to get health checks, participate in community-based healthy lifestyle activities, and engage in programs to manage their type 2 diabetes [54,55]. On the other hand, common infectious diseases (e.g., gastrointestinal, respiratory, acute rheumatic fever) in remote Indigenous communities have been associated with unhealthy and non-functional residential housing infrastructure [49,56]. For example, lack of quality water supply to and broken toilets and taps can preclude remote community households to maintain hygiene to protect from the development and transmission of acute rheumatic fever and diarrheal diseases.

These findings can be contextualized in the current COVID-19 pandemic scenario, where access to a continuous water supply and adequate residential housing infrastructure are considered essential prerequisites for ensuring good sanitation and hygiene practices [30]. Inadequate or faulty home hardware, overcrowding, and lack of housing repairs and maintenance, can make it challenging for communities to respond to and recover from infectious disease outbreaks [25]. Issues related to the access and utilization of health services during the pandemic could also be much worse for remote and rural communities than for regional and urban communities [57,58]. A few studies highlight the lack of adequate transportation facilities for Indigenous people to access health services, which is a well-established key barrier to patient management and continued illness care in remote Indigenous communities in Australia [59,60].

Access to local health services is essential to provide clinical support and promote public health messages related to support physical/social distancing, hand washing and isolation requirements during disease outbreaks. Therefore improvements to residential environmental living conditions, and access to local health services (supported through strengthened transportation and communication networks) can play an important role during disease outbreaks like COVID-19, which are often unpredictable [30,61]. Despite the fact that infections due to viral and bacterial pathogens as well as protozoan parasites have been identified as a problem by clinicians and epidemiologists more broadly, little attention has been given to identifying which environmental determinants influence such infectious diseases in Australia, particularly in remote Indigenous communities. Some epidemiological studies, however, have examined the association between infectious disease outcomes and environmental transmission pathways such as housing, water and sanitation, and access to health care at the wider community level [56,62,63].

The ‘community vitality’ region comprised the clusters of (1) community economic resources, and (2) community services. The ‘community economic resources’ cluster includes relevant features of remote community environments such as agricultural and processing operations (ID# 39), local mining operations (ID# 82), open space tourist accommodation (ID# 78) and protection of sacred sites (ID# 42). However, in an Indigenous context the indicators within this cluster highlight deep historical contentions [64,65]. For example there are instances where Indigenous peoples are likely to experience the burden from resource-intensive and resource-extractive industries, such as mining, industrial fishing and farming, eco-tourism and imposed conservation projects impacting the protection of their important cultural sacred sites [65]. These extractive industries undermine Indigenous peoples’ self-determination and ability, in some instances, to fulfill their cultural obligations and care for country. The ‘community services’ cluster includes features, such as retail shopping facilities (ID# 1) and general business services (ID# 26). In urban contexts the availability of supermarkets and groceries, as a source of healthful food environments, is strongly associated with lower body weight, while access to convenience stores is a predictor of higher body weight [66]. Lower prices for vegetable and fruits, and increased prices for fast-food meals, is also associated with significantly lower BMI [67,68]. These findings are conceptually relevant for remote Indigenous communities in Australia which are often limited to a single local community store with significantly higher prices for healthful foods, and cheaper prices for fast foods [69]. Pricing and disruption in supply of healthful foods can also be exacerbated by unreliable power supply for stores to preserve perishables, when such food items cannot be delivered during wet season due to road cut-off. This can have a significant influence on people’s choosing cheaper fast foods and developing chronic disease [70].

The ‘protecting and maintaining natural environments’ region comprised the sole cluster ‘environmental protection and climate change’. Built indicators related to the ‘environmental protection and climate change’ include features, such as environmental protection from mining (ID# 16), energy and water conservation (ID# 37, and 45), alternative renewable energy sources (ID# 59), and environmental education centers (ID# 53). Existing research has highlighted the importance of environmental conservation, dust control, and readjustment of housing infrastructure for remote communities in Australia to adapt to the challenges of climate change [51,71]. These studies predict immense challenges for remote Indigenous communities in the NT due to extreme weather patterns, such as increasing temperatures, and incidence of frequent cyclones and flash flooding. The indicators rated as important within the ‘environmental protection and climate change’ cluster may inform future housing infrastructure development to support comfort within households, and influence family health and wellbeing preventing chronic disease [72,73].

### 4.1. Implications for Public and Environmental Health Policy and Practice

In this GCM study, the frontline, managerial and policy-level workforce identified and prioritized the indicators as most important for both chronic and infectious diseases. Within the NT Public Health and Environmental Health portfolio, workforce members are responsible for ensuring that business and community facilities uphold minimum environmental health standards [74]. These standards capture features of environments such as housing, public sanitation facilities, water supply, power supply, sewerage systems, rubbish collection and disposal, repair and maintenance of environmental health infrastructure. Systematically selected built environmental indicators generated in the scoping review [43], followed by this concept mapping study, may support the environmental and public health sectors to identify priority indicators that can be actioned in the shorter term (e.g., within the next 2–3 years) and the longer term (within 5–10 years). The most important built environmental indicators identified in this research may provide an evidence base for the NT Department of Health to influence the broader government system through networking, communicating, and negotiating (on behalf of the community) with regional councils and other sections in the government system to address local-level environmental health issues.

### 4.2. Strengths

Applying an iKT approach, engaging Indigenous and non-Indigenous stakeholders in all steps of the GCM process was a key strength of this study. It ensured the relevance of research outcomes and, the likelihood that the research outcomes would inform public and environmental health practice [34]; and that important contextual and cultural aspects of the local community would not be overlooked [38]. Project objectives were adjusted to reflect stakeholder interests to include both infectious disease and chronic disease. A senior policy officer from the NT Department of Environmental Health (N.S.) was an associate investigator on the EnRICH project, in which this GCM study was nested, to enable timely access to research results and inform environmental health policy actions.

The brainstorming component of the concept mapping study identified locally relevant built environmental indicators from a scoping review of community and strategic planning documents [43] with evidence of Indigenous input [75]. In our discussions, stakeholders identified several policy and strategic planning documents developed through community consultation. These documents included community-level environmental factors relevant to the development of chronic disease and more broadly to community health and wellbeing. Therefore, to capture community perspectives in existing documents, and to avoid burdening communities with additional consultation to brainstorm ‘de novo’ built environmental indicators, a scoping review of such under-utilized publicly available documents [43] was undertaken to identify and consolidate indicators for this GCM study.

### 4.3. Limitations

The first limitation pertains to sample characteristics. While this study was successful in gaining the perspectives from the frontline and policy-level workforce, the majority of these participants were from the government sector and were non-Indigenous. Future concept mapping studies should attempt to recruit more participants identifying as Indigenous and with experience working in the Indigenous community-controlled sector. The small sample size and under-representation of Indigenous stakeholders influences the generalizability of the findings as such may not be transferable to other remote Indigenous communities beyond the NT. Future concept mapping studies should consider a carefully crafted sampling plan geared towards recruiting participants with greater experience for the sorting exercise.

Second, the final concept map represented a ‘best fit’ by aggregating the sorting results from the 20 participants, yet it is not clear that the ‘best fit’ was achieved. For instance, there were Indicator statement(s) within clusters, which ideally should have been grouped, in a different cluster. One specific example is, ‘access to road ambulance and other emergency transportation facilities’ (ID# 34) from the ‘municipal and emergency services’ cluster, which may have been better suited for the ‘facilities for health and safety’ cluster. Statements can only be re-allocated to adjacent clusters to preserve the condition of non-overlapping. That a ‘best fit’ may not have been achieved reflect, to some extent, the complexity of the topic and the interpretations that were given by participants to their ‘piles’ during the sorting task.

## 5. Conclusions

The 9-cluster solution concept map reflects aggregated perceptions of stakeholder participants with greater than 6 years of experience working in the government and non-government sector in the NT. The ‘essential services’ and ‘facilities for health and safety’ clusters had the highest perceived importance ratings for infectious disease and chronic disease respectively. The results from this study can inform public health planning efforts by identifying priority areas to guide the systematic collection of indicators to monitor progress of health service provision aimed at improving environmental living conditions and Closing the Gap in remote Indigenous communities. As a policy implication, knowing which aspects of community environments are perceived as most important to the development of diseases is essential, as actions can then be taken to address those unhealthful aspects of the environments. The findings from this research highlight the need for future epidemiological research investigating the association between the features of remote Indigenous community environments in the NT. The built environmental indicators that emerged in the ‘Go-Zone’ analysis may provide the basis to undertake such inferential analysis.

## Figures and Tables

**Table 1:** Demographic characteristics of participants in the sorting and rating activities.

Categories	Sorting (*n* = 29)	Rating (*n* = 42)
Type of organization		
Government	26 (90%)	36 (85%)
Aboriginal and Torres Strait Islander Community Controlled	3 (10%)	4 (10%)
Other non-government organization	0 (0%)	2 (5%)
Areas of work		
NT mostly remote	18 (62%)	24 (57%)
NT mostly rural	4 (14%)	5 (12%)
NT mostly urban	7 (24%)	9 (21%)
Other remote	0 (0%)	4 (10%)
Indigenous status		
Aboriginal	9 (31%)	10 (24%)
Neither Aboriginal or Torres Strait Islander	20 (69%)	32 (76%)
Duration in the position		
<6 years	9 (31%)	13 (31%)
6–9 years	2 (7%)	5 (12%)
≥10 years	18 (62%)	24 (57%)
Position or role		
Frontline worker	18 (62%)	25 (60%)
Project Officer	4 (14%)	4 (10%)
Program Manager	1 (3%)	6 (14%)
Policy Officer	2 (7%)	2 (5%)
Other managerial or policy level position	4 (14%)	5 (11%)

## Data Availability

Not applicable.

## Supplementary Materials

The following are available online at https://www.mdpi.com/article/10.3390/ijerph18105178/s1, Table S1: Indicator statements with accompanying mean importance ratings (chronic disease and infectious disease), combined average importance ratings (chronic disease and infectious disease), and bridging values, by cluster.

## References

[1] Prüss-Üstün A., Wolf J., Corvalán C., Bos R., Neira M. (2016). Preventing Disease through Healthy Environments: A Global Assessment of the Burden of Disease from Environmental Risks.

[2] Aboriginal and Torres Strait Islander Social Justice Commissioner (2005). Social Justice Report 2005.

[3] Aboriginal and Torres Strait Islander Social Justice Commissioner (2015). Social Justice and Native Title Report 2015.

[4] Paradies Y., Cunningham J. (2002). Placing Aboriginal and Torres Strait Islander Mortality in an International Context. Aust. N. Z. J. Public Health.

[5] Anderson I., Crengle S., Kamaka M.L., Chen T.-H., Palafox N., Jackson-Pulver L. (2006). Indigenous Health in Australia, New Zealand, and the Pacific. Lancet.

[6] Australian Institute of Health and Welfare (2015). The Health and Welfare of Australia’s Aboriginal and Torres Strait IslanderPeoples: 2015.

[7] Vos T., Barker B., Begg S., Stanley L., Lopez A.D. (2009). Burden of Disease and Injury in Aboriginal and Torres Strait Islander Peoples: The Indigenous Health Gap. Int. J. Epidemiol..

[8] Australian Institute of Health and Welfare (2014). Mortality Inequalities in Australia 2009–2011.

[9] Zhao Y., You J., Wright J., Guthridge S.L., Lee A.H. (2013). Health Inequity in the Northern Territory, Australia. Int. J. Equity Health.

[10] Gracey M., King M. (2009). Indigenous Health Part 1: Determinants and Disease Patterns. Lancet.

[11] Saggers S., Gray D. (1991). Aboriginal Health and Society: The Traditional and Contemporary Aboriginal Struggle for Better Health.

[12] Daniel M., Lekkas P., Cargo M., Stankov I., Brown A. (2011). Environmental Risk Conditions and Pathways to Cardiometabolic Diseases in Indigenous Populations. Annu. Rev. Public Health.

[13] Gray C., Brown A., Thomson N. (2012). Review of Cardiovascular Health among Indigenous Australians. Diabetes Care.

[14] Ejere H., Alhassan M.B., Rabiu M. (2004). Face Washing Promotion for Preventing Active Trachoma. Cochrane Database Syst. Rev..

[15] McDonald E., Bailie R., Brewster D., Morris P. (2008). Are Hygiene and Public Health Interventions Likely to Improve Outcomes for Australian Aboriginal Children Living in Remote Communities? A Systematic Review of the Literature. BMC Public Health.

[16] Rose G. (2001). Sick Individuals and Sick Populations. Int. J. Epidemiol..

[17] Marmot M. (2015). The Health Gap: The Challenge of an Unequal World. Lancet.

[18] Australian Institute of Health and Walfare (2011). Health and the Environment: A Compilation of Evidence.

[19] Frank L.D., Saelens B.E., Powell K.E., Chapman J.E. (2007). Stepping Towards Causation: Do Built Environments or Neighborhood and Travel Preferences Explain Physical Activity, Driving, and Obesity?. Soc. Sci. Med..

[20] Saelens B.E., Sallis J.F., Frank L.D. (2003). Environmental Correlates of Walking and Cycling: Findings from the Transportation, Urban Design, and Planning Literatures. Ann. Behav. Med..

[21] Høj S.B., Paquet C., Caron J., Daniel M. (2021). Relative ‘Greenness’ and not Availability of Public Open Space Buffers Stressful Life Events and Longitudinal Trajectories of Psychological Distress. Health Place.

[22] Carroll S.J., Dale M.J., Taylor A.W., Daniel M. (2020). Contributions of Multiple Built Environment Features to 10-Year Change in Body Mass Index and Waist Circumference in a South Australian Middle-Aged Cohort. Int. J. Environ. Res. Public Health.

[23] Gal C.L., Dale M.J., Cargo M., Daniel M. (2020). Built Environments and Cardiometabolic Morbidity and Mortality in Remote Indigenous Communities in the Northern Territory, Australia. Int. J. Environ. Res. Public Health.

[24] Carroll S.J., Turrell G., Dale M.J., Daniel M. (2021). Associations between Supermarket Availability and Body Sze in Australia: A Cross-sectional Observational Study Comparing State and Territory Capital Cities. BMC Public Health.

[25] Ali S.H., Foster T., Hall N.L. (2018). The Relationship between Infectious Diseases and Housing Maintenance in Indigenous Australian Households. Int. J. Environ. Res. Public Health.

[26] Coffey P.M., Ralph A.P., Krause V.L. (2018). The Role of Social Determinants of Health in the Risk and Prevention of Group A Streptococcal Infection, Acute Rheumatic Fever and Rheumatic Heart Disease: A Systematic Review. PLoS Negl. Trop. Dis..

[27] Edmond K., Scott S., Korczak V., Ward C., Sanderson C., Theodoratou E., Clark A., Griffiths U., Rudan I., Campbell H. (2012). Long Term Sequelae from Childhood Pneumonia; Systematic Review and Meta-analysis. PLoS ONE.

[28] Cunningham M.W. (2003). Autoimmunity and Molecular Mimicry in the Pathogenesis of Post-streptococcal Heart Disease. Front. Biosci..

[29] O’Neill J. (2016). Tackling Drug-resistant Infections Globally: Final Report and Recommendations: Review on Antimicrobial Resistance.

[30] Department of Health (2020). Australian Health Sector Emergency Response Plan for Novel Corronavirus (COVID-19): Management and Operational Plan for Aborginal and Torres Strait Islander Populations Canberra: Australian Government. https://www.health.gov.au/sites/default/files/documents/2020/07/management-plan-for-aboriginal-and-torres-strait-islander-populations.pdf.

[31] Productivity Commission (2020). Indigenous Evaluation Strategy.

[32] Australian Institute of Health and Welfare (2004). Rural Regional and Remote Health: A Guide to Remoteness Classifications.

[33] ABS (2016). 2016 Census of Population and Housing, Northern Territory: Aboriginal and Torres Strait Islander Peoples Profile: Australian Bureau of Statistics (ABS). https://www.abs.gov.au/websitedbs/D3310114.nsf/Home/2016%20search%20by%20geography.

[34] Graham I.D., Kothari A., McCutcheon C. (2018). Moving Knowledge into Action for more Effective Practice, Programmes and Policy: Protocol for a Research Programme on Integrated Knowledge Translation. Implement. Sci..

[35] Kothari A., McCutcheon C., Graham I.D. (2017). Defining Integrated Knowledge Translation and Moving Forward: A Response to Recent Commentaries. Int. J. Health Policy Manag..

[36] Trochim W.M., McLinden D. (2017). Introduction to a Special Issue on Concept Mapping. Eval. Program Plan..

[37] Trochim W.M. (2017). Hindsight is 20/20: Reflections on the Evolution of Concept Mapping. Eval. Program Plan..

[38] Vaughn L.M., Jones J.R., Booth E., Burke J.G. (2017). Concept Mapping Methodology and Community-Engaged Research: A Perfect Pairing. Eval. Program Plan..

[39] Kane M., Trochim W.M. (2007). Concept Mapping for Planning and Evaluation.

[40] Dawson A.P., Cargo M., Stewart H., Chong A., Daniel M. (2012). Aboriginal Health Workers Experience Multilevel Barriers to Quitting Smoking: A Qualitative Study. Int. J. Equity Health.

[41] Stankov I., Howard N., Daniel M., Cargo M. (2017). Policy, Research and Residents’ Perspectives on Built Environments Implicated in Heart Disease: A Concept Mapping Approach. Int. J. Environ. Res. Public Health.

[42] Cargo M., Potaka-Osborne G., Cvitanovic L., Warner L., Clarke S., Judd J., Chakraborty A., Boulton A. (2019). Strategies to Support Culturally Safe Health and Wellbeing Evaluations in Indigenous Settings in Australia and New Zealand: A Concept Mapping Study. Int. J. Equity Health.

[43] Chakraborty A., Daniel M., Howard N.J., Chong A., Slavin N., Brown A., Cargo M. (2021). Identifying Environmental Determinants Relevant to Health and Wellbeing in Remote Australian Indigenous Communities: A Scoping Review of Grey Literature. Int. J. Environ. Res. Public Health.

[44] Marks E., Cargo M.D., Daniel M. (2007). Constructing a Health and Social Indicator Framework for Indigenous Community Health Research. Soc. Indic. Res..

[45] Daniel M., Cargo M., Marks E., Paquet C., Simmons D., Williams M., Rowley K., O’Dea K. (2009). Rating Health and Social Indicators for Use with Indigenous Communities: A Tool for Balancing Cultural and Scientific Utility. Soc. Indic. Res..

[46] Kruskal J.B., Wish M. (1978). Multidimensional Scaling.

[47] Lebel A., Cantinotti M., Pampalon R., Thériault M., Smith L.A., Hamelin A.-M. (2011). Concept Mapping of Diet and Physical Activity: Uncovering Local Stakeholders Perception in the Quebec City Region. Soc. Sci. Med..

[48] Rosas S.R., Kane M. (2012). Quality and Rigor of the Concept Mapping Methodology: A Pooled Study Analysis. Eval. Pogram Plan..

[49] Bailie R.S., Wayte K.J. (2006). Housing and Health in Indigenous Communities: Key Issues for Housing and Health Improvement in Remote Aboriginal and Torres Strait Islander Communities. Aust. J. Rural. Health.

[50] McMullen C., Eastwood A., Ward J. (2015). Environmental Attributable Fractions in Remote Australia: The Potential of a New Approach for Local Public Health Action. Aust. N. Z. J. Public Health.

[51] Melody S., Bennett E., Clifford H., Johnston F., Shepherd C., Alach Z., Lester M., Wood L.J., Franklin P., Zosky G.R. (2016). A Cross-sectional Survey of Environmental Health in Remote Aboriginal Communities in Western Australia. Int. J. Environ. Health Res..

[52] Foster T., Hall N.L. (2021). Housing Conditions and Health in Indigenous Australian Communities: Current Status and Recent Trends. Int. J. Environ. Health Res..

[53] Bailie R.S., Stevens M., McDonald E.L. (2012). The Impact of Housing Improvement and Socio-environmental Factors on Common Childhood Illnesses: A Cohort Study in Indigenous Australian Communities. J. Epidemiol. Community Health.

[54] Bailie C., Matthews V., Bailie J., Burgess P., Copley K., Kennedy C., Moore L., Larkins S., Thompson S., Bailie R.S. (2016). Determinants and Gaps in Preventive Care Delivery for Indigenous Australians: A Cross-sectional Analysis. Front. Public Health.

[55] Ware V.-A. (2013). Improving the Accessibility of Health Services in Urban and Regional Settings for Indigenous People.

[56] Roberts K.V., Maguire G.P., Brown A., Atkinson D.N., Remenyi B., Wheaton G., Ilton M., Carapetis J. (2015). Rheumatic Heart Disease in Indigenous Children in Northern Australia: Differences in Prevalence and the Challenges of Screening. Med. J. Aust..

[57] Crooks K., Casey D., Ward J. (2020). First Nations People Leading the Way in COVID-19 Pandemic Planning, Response and Management. Med. J. Aust..

[58] Moss R., Wood J., Brown D., Shearer F., Black A.J., Cheng A., McCaw J.M., McVernon J. (2020). Modelling the Impact of COVID-19 in Australia to Inform Transmission Reducing Measures and Health System Preparedness. medRXiv.

[59] Artuso S., Cargo M., Brown A., Daniel M. (2013). Factors Influencing Health Care Utilisation among Aboriginal Cardiac Patients in Central Australia: A Qualitative Study. BMC Health Serv. Res..

[60] Bailie J., Schierhout G., Laycock A., Kelaher M., Percival N., O’Donoghue L., McNeair T., Bailie R. (2015). Determinants of Access to Chronic Illness Care: A Mixed-methods Evaluation of a National Multifaceted Chronic Disease Package for Indigenous Australians. BMJ Open.

[61] Price Waterhouse Coopers (2007). Indigenous Housing: Findings of the Review of the Community Housing and Infrastructure Programme.

[62] Bailie R.S., Stevens M.R., McDonald E., Halpin S., Brewster D., Robinson G., Guthridge S. (2005). Skin Infection, Housing and Social Circumstances in Children Living in Remote Indigenous Communities: Testing Conceptual and Methodological Approaches. BMC Public Health.

[63] Holt D.C., McCarthy J.S., Carapetis J.R. (2010). Parasitic Diseases of Remote Indigenous Communities in Australia. Int. J. Parasitol..

[64] Jacklin K. (2009). Diversity within: Deconstructing Aboriginal Community Health in Wikwemikong Unceded Indian Reserve. Soc. Sci. Med..

[65] United Nations (2009). State of the World’s Indigenous Peoples.

[66] Leal C., Chaix B. (2011). The Influence of Geographic Life Environments on Cardiometabolic Risk Factors: A Systematic Review, a Methodological Assessment and a Research Agenda. Obes. Rev..

[67] Powell L.M., Auld M.C., Chaloupka F.J., O’Malley P.M., Johnston L.D. (2006). Access to Fast Food and Food Prices: Relationship with Fruit and Vegetable Consumption and Overweight among Adolescents. The Economics of Obesity.

[68] Sturm R., Datar A. (2005). Body Mass Index in Elementary School Children, Metropolitan Area Food Prices and Food Outlet Density. Public Health.

[69] Brimblecombe J.K., Ferguson M.M., Liberato S.C., O’Dea K. (2013). Characteristics of the Community-level Diet of Aboriginal People in Remote Northern Australia. Med. J. Aust..

[70] Henryks J., Brimblecombe J. (2016). Mapping Point-of-Purchase Influencers of Food Choice in Australian Remote Indigenous Communities. SAGE Open.

[71] Race D., Mathew S., Campbell M., Hampton K. (2016). Understanding Climate Adaptation Investments for Communities Living in Desert Australia: Experiences of Indigenous Communities. Clim. Chang..

[72] Turner L.R., Connell D., Tong S. (2013). The Effect of Heat Waves on Ambulance Attendances in Brisbane, Australia. Prehospital Disaster Med..

[73] Momperousse D., Delnevo C., Lewis M. (2007). Exploring the Seasonality of Cigarette-smoking Behaviour. Tob. Control..

[74] Department of Health (2001). Environmental Health Standards for Remote Communities in the Northern Territory Darwin: Department of Health. http://digitallibrary.health.nt.gov.au/prodjspui/handle/10137/874?mode=full.

[75] Fraser E.D.G., Dougill A.J., Mabee W.E., Reed M., McAlpine P. (2006). Bottom Up and Top Down: Analysis of Participatory Processes for Sustainability Indicator Identification as a Pathway to Community Empowerment and Sustainable Environmental Management. J. Environ. Manag..

