# Supplementary material for: Prioritizing Built Environmental Factors to Tackle Chronic and Infectious Diseases in Remote Northern Territory (NT) Communities of Australia: A Concept Mapping Study
Source: Int J Environ Res Public Health. 2021 May 13;18(10):5178. doi: 10.3390/ijerph18105178 (PMC8152969; doi:10.3390/ijerph18105178)
Supplement: Supplementary file 1 [file ijerph-18-05178-s001.zip › ijerph-1202358-SI.pdf]

Supplementary Materials: Prioritizing built environmental factors to tackle their influence on chronic and infectious diseases in remote Northern Territory (NT) communities of Australia: a concept mapping study

Amal Chakraborty, Natasha J. Howard, Mark Daniel, Alwin Chong, Nicola Slavin, Alex Brown, and Margaret Cargo

Table S1. Indicator statements with accompanying mean importance ratings (chronic disease and infectious disease), combined average importance ratings (chronic disease and infectious disease), and bridging values, by cluster.

ID	Statement		ICD	IID	Avg ICD and IID	BV
Cluster 1: Community services		Relative representation of statements in the 'Go-Zone' (40%)	3.16	2.98	3.07	0.46
1	* retail shopping facilities including community store		4.07	3.52	3.80	0.37
5	* up-to-date health technologies equipment		4.40	4.33	4.37	0.39
10	banking and postal service facilities		2.55	2.26	2.41	0.43
26	general business services (including mechanic)		2.69	2.48	2.59	0.59
81	commercial visitor accommodation (including hotel, motel and guest house)		2.10	2.29	2.20	0.50
Cluster 2: Facilities for education and culture		Relative representation of statements in the 'Go-Zone' (30%)	3.25	3.12	3.19	0.50
14	access to school facilities for after hour sports		3.40	2.79	3.10	0.52
19	* access to primary school		3.76	3.74	3.75	0.64
27	community public library facilities		2.26	2.26	2.26	0.43
28	* access to secondary school		3.81	3.74	3.78	0.57
31	early learning centres (including child care centre/creche, pre-school)		3.29	3.71	3.50	0.43
41	vocational education and training centre (e.g. TAFE, trade training centre)		3.36	3.14	3.25	0.48
56	community arts, crafts, language and cultural centres		3.12	2.93	3.03	0.52
57	community markets		2.46	2.17	2.32	0.52
60	* community-based adult education centre		3.43	3.50	3.47	0.44
76	access to culturally appropriate men's and women's shed		3.60	3.24	3.42	0.51

ID	Statement		ICD	IID	Avg ICD and IID	BV
Cluster 3: Facilities for health and safety		Relative representation of statements in the 'Go-Zone' (67%)	4.02	3.90	3.96	0.43
4	* adequate residential housing (including condition and overcrowding)		4.64	4.81	4.73	0.43
6	* access to hospital services and birthing facilities		4.62	4.60	4.61	0.34
11	access to safe house facilities (e.g. refuge, crisis shelter)		3.21	3.07	3.14	0.35
46	access to facilities for people with special needs (e.g. disable ramp)		4.07	3.12	3.60	0.54
49	* adequate staff housing (including health clinic, teachers, government, and other organisation's staff members)		3.62	3.37	3.50	0.52
65	adequate visitor/contractor's accommodation		2.86	3.05	2.96	0.53
67	* laundromat facilities		3.81	4.24	4.03	0.35
74	* community health centre providing primary health care, allied health and specialist services		4.83	4.67	4.75	0.45
79	* aged care, respite centre or disability service facilities		4.50	4.17	4.34	0.42
Cluster 4: Sports and recreational facilities		Relative representation of statements in the 'Go-Zone' (15%)	3.12	2.80	2.96	0.49
7	children's playground		3.37	2.74	3.06	0.45
8	sporting facilities including football club, oval facilities, basketball court, tennis court		3.90	3.05	3.48	0.48
22	skate park infrastructure		2.26	1.90	2.08	0.55
29	* swimming pools and waterparks		3.49	3.48	3.49	0.53
58	multipurpose community hall		2.95	2.74	2.85	0.35
66	landscaping and revegetation of parks, gardens and reserves		2.79	2.76	2.78	0.59
68	parks, gardens and reserves infrastructure (including park furniture, shade, and other facilities)		3.07	2.93	3.00	0.52
Cluster 5: Essential services		Relative representation of statements in the 'Go-Zone' (67%)	3.73	4.12	3.93	0.08
2	fencing around existing essential service infrastructure		2.62	3.24	2.93	0.04
9	* reliable sewerage system		4.24	4.88	4.56	0.04
13	* stormwater and drainage system		3.55	4.24	3.90	0.04
15	* maintenance of water system infrastructure		4.34	4.76	4.55	0.02
20	* regular rubbish collection from households and public places		3.79	4.62	4.21	0.02
23	* access to continuous water supply		4.74	4.88	4.81	0.02

ID	Statement	ICD	IID	Avg ICD and IID	BV
24	* availability of community infrastructure maintenance services (e.g. general repairs, electrical)	4.12	4.12	4.12	0.02
30	* pest control equipment and facilities	3.45	4.21	3.83	0.16
36	* access to power services	4.43	4.19	4.31	0.00
40	public toilet facilities	3.29	3.81	3.55	0.19
43	* availability of housing infrastructure maintenance services (e.g. general repairs, electrical)	4.33	4.57	4.45	0.22
50	lighting for streets and outdoor buildings	3.07	2.74	2.91	0.08
52	animal management equipment and facilities	3.24	4.00	3.62	0.23
61	availability of municipal services plant equipment (e.g. works depot, council vehicle fleets)	3.00	3.36	3.18	0.07
70	recycling of rubbish and hard waste (including steel, car, electronic equipment)	2.79	3.24	3.02	0.16
77	* management and protection of landfills and dumpsites	3.43	4.12	3.78	0.05
83	* reliable power infrastructure	4.43	4.46	4.45	0.01
84	* adequate septic system	4.36	4.78	4.57	0.04
Cluster 6: Municipal and emergency services	Relative representation of statements in the 'Go-Zone' (46%)	3.29	3.17	3.23	0.24
12	construction and maintenance of community roads	3.19	2.88	3.04	0.08
17	emergency public shelter and disaster management services	2.93	3.62	3.28	0.12
25	* access to emergency air transportation facilities (e.g. Royal Flying Doctor Service)	4.52	4.14	4.33	0.29
32	footpaths, bike paths and walkways	2.90	2.38	2.64	0.29
33	adequate traffic safety and speed restrictions (including road signage)	2.43	2.20	2.32	0.35
34	* access to road ambulance and other emergency transportation facilities	4.12	3.88	4.00	0.36
38	access to cemetery and grave shoring system	2.66	2.93	2.80	0.34
54	* access to air transport facilities (including aircrafts, wheelchair lift, runway, and aerodrome)	3.76	3.43	3.60	0.16
55	* appropriate land development (including community infrastructure and new housing)	3.81	4.02	3.92	0.21
62	construction and maintenance of highways	3.10	2.81	2.96	0.10
69	* access to fuel	3.79	3.38	3.59	0.27
71	public vehicle spaces	1.95	2.05	2.00	0.31
72	* Government service centre facilities (e.g. Centrelink, housing, and business services)	3.64	3.45	3.55	0.23

ID	Statement	ICD	IID	Avg ICD and IID	BV
Cluster 7: Transportation and communication	Relative representation of statements in the 'Go-Zone' (0%)	3.12	2.89	3.01	0.40
3	remote area radio broadcasting facilities	2.62	2.55	2.59	0.47
18	up-to-date telecommunication technologies (including ICT equipment, wireless and broadband network)	3.38	3.24	3.31	0.39
35	reliable mobile phone infrastructure	3.57	3.29	3.43	0.26
44	access to bus transport facilities between communities	3.12	2.74	2.93	0.61
47	access to water transport facilities (including boats, ferries, wharfs, barge landing, pontoons)	2.44	2.45	2.45	0.47
63	access to telephone service facilities in residential households	3.12	2.88	3.00	0.40
73	remote area tv broadcasting facilities	3.14	3.12	3.13	0.36
75	access to local community bus transport facilities	3.14	2.71	2.93	0.40
80	access to public telephone facilities	3.52	3.02	3.27	0.27
Cluster 8: Environmental protection and climate change	Relative representation of statements in the 'Go-Zone' (0%)	2.66	2.78	2.72	0.64
16	environmental protection from mining operations	3.21	3.29	3.25	0.74
21	natural environmental protection (including water bodies, reducing the ocean pollution and biodiversity management)	3.29	3.57	3.43	0.66
37	energy conservation	2.48	2.43	2.46	0.60
45	water conservation	2.95	3.12	3.04	0.59
51	impact of climate change (e.g. mitigating the impact of tidal inundation and erosion)	2.43	2.67	2.55	0.60
53	environmental education centre (e.g. incorporating threatened species research)	2.67	2.83	2.75	0.82
59	access to alternative and renewable energy resources	2.50	2.40	2.45	0.50
64	sea wall infrastructure	1.71	1.90	1.81	0.60
Cluster 9: Community economic resources	Relative representation of statements in the 'Go-Zone' (0%)	2.51	2.39	2.45	0.81
39	agricultural and processing operations (fruits, vegetable farms, piggery, chicken farms, meat works)	2.95	2.88	2.92	0.71
42	access to and protection of important sacred sites or culturally significant places (e.g. cultural zone and heritage park)	3.17	2.76	2.97	1.00
48	fishing operations and processing plants	2.10	2.00	2.05	0.82

ID	Statement	ICD	IID	Avg ICD and IID	BV
78	open space tourist accommodation (including caravan park, camping ground, holiday park and resort etc.)	1.93	1.98	1.96	0.77
82	local mining operations	2.40	2.34	2.37	0.77

ICD = importance rating chronic disease; IID = importance rating infectious disease; Avg ICD and IID = combined average importance ratings for chronic and infectious diseases; BV = bridging value; * = statements in the 'Go-Zone'.
